# Glyoxalase 1-knockdown in human aortic endothelial cells – effect on the proteome and endothelial function estimates

**DOI:** 10.1038/srep37737

**Published:** 2016-11-29

**Authors:** Bernd Stratmann, Britta Engelbrecht, Britta C. Espelage, Nadine Klusmeier, Janina Tiemann, Thomas Gawlowski, Yvonne Mattern, Martin Eisenacher, Helmut E. Meyer, Naila Rabbani, Paul J. Thornalley, Diethelm Tschoepe, Gereon Poschmann, Kai Stühler

**Affiliations:** 1 Herz und Diabeteszentrum NRW, Diabeteszentrum, Ruhr-Universität Bochum, Bad Oeynhausen, Germany; 2 Medical Proteom-Center, Ruhr-Universität Bochum, Bochum, Germany; 3 Warwick Medical School, Clinical Sciences Research Laboratories, University of Warwick, University Hospital, Coventry UK; 4 Molecular Proteomics Laboratory, BMFZ, Heinrich-Heine-University, Düsseldorf, Germany; 5 Institute for Molecular Medicine, Heinrich-Heine-University, Düsseldorf, Germany

## Abstract

Methylglyoxal (MG), an arginine-directed glycating agent, is implicated in diabetic late complications. MG is detoxified by glyoxalase 1 (GLO1) of the cytosolic glyoxalase system. The aim was to investigate the effects of MG accumulation by GLO1-knockdown under hyperglycaemic conditions in human aortic endothelial cells (HAECs) hypothesizing that the accumulation of MG accounts for the deleterious effects on vascular function. SiRNA-mediated knockdown of GLO1 was performed and MG concentrations were determined. The impact of MG on the cell proteome and targets of MG glycation was analysed, and confirmed by Western blotting. Markers of endothelial function and apoptosis were assessed. Collagen content was assayed in cell culture supernatant. GLO1-knockdown increased MG concentration in cells and culture medium. This was associated with a differential abundance of cytoskeleton stabilisation proteins, intermediate filaments and proteins involved in posttranslational modification of collagen. An increase in fibrillar collagens 1 and 5 was detected. The extracellular concentration of endothelin-1 was increased following GLO1-knockdown, whereas the phosphorylation and amount of eNOS was not influenced by GLO1-knockdown. The expression of ICAM-1, VCAM-1 and of MCP-1 was elevated and apoptosis was increased. MG accumulation by GLO1-knockdown provoked collagen expression, endothelial inflammation and dysfunction and apoptosis which might contribute to vascular damage.

Endothelial dysfunction (ED)^1^ and low-grade inflammation may partially explain the increased risk for cardiovascular disease (CVD) in patients with diabetes mellitus^2^,^3^. Promoters of ED are reactive oxygen species (ROS), either resulting from glucotoxicity or lipotoxicity, hypertension, insulin resistance, elevated levels of adhesive molecules, disturbances in vasorelaxation and -constriction, and dicarbonyl stress decreasing NO bioavailability^2^,^4^,^5^,^6^,^7^. The latter is defined as imbalance of reactive dicarbonyl glycating agents and enzyme activities leading to increased protein glycation^8^. A major dicarbonyl metabolite accumulating in diabetes is methylglyoxal (MG), the concentration in blood is increased 4–5fold in patients with type 1 and 2–3fold in patients with type 2 diabetes^9^. MG is formed by degradation of triosephosphates (Fig. 1A) with usually minor formation from ketone body metabolism, threonine catabolism and degradation of glycated proteins. MG is a potent glycating agent and precursor of advanced glycation endproducts (AGEs) of proteins. It reacts mainly with arginine residues to form hydroimidazolone MG-H1 (Fig. 1B). This often leads to dysfunction because arginine residues, particularly those susceptible to modification, are found at functional sites of proteins^10^. Increased levels of MG-H1 are detected in diabetes^11^,^12^.

MG is efficiently metabolised and maintained at low levels mainly by glyoxalase 1 (GLO1) of the glutathione-dependent glyoxalase system in the cytosol of all cells (Fig. 1C)^9^. This detoxifying system is downregulated by increased MG formation and decreased GLO1 activity in endothelial cells cultured under long term high glucose concentration as a model of hyperglycaemic conditions^13^,^14^. Increased MG concentration in cells and culture medium leads to increased formation of MG-H1 in integrin binding sites of collagen type-IV, resulting in endothelial cell detachment and anoikis^15^. Increased glycation of cellular proteins is likely linked to increased formation of ROS and inflammatory signalling^14^,^16^. Conversely, inhibition of GLO1 with a specific GLO1 inhibitor, S-p-bromobenzylglutathione cyclopentyl diester (BBGSHCp_2_) led to accumulation of MG in low glucose concentration and dysfunction similar to that found in the hyperglycaemia model^15^. Increasing dicarbonyl stress in the absence of hyperglycaemia was detected in mice by administration of BBGSHCp_2_ or exogenous MG increased endothelial inflammation and atherogenesis similar to that observed in diabetes^17^.

These findings lead to the suggestion that deleterious actions of hyperglycaemia may be related to increased concentration of MG caused by increased formation and decreased detoxification.

MG has been identified as major precursor for AGEs in aortic endothelial cells and aortal extracellular matrix^15^. Decreased formation of MG-derived AGEs by GLO1 overexpression prevents ED in experimental diabetes with concomitant improvement of vascular function^6^. Furthermore, overexpression of GLO1 prevented accumulation of MG-H1 in streptozocin-induced diabetes as well as loss of endothelium-dependent reactivity in mesenteric arteries^18^. Down regulation of GLO1 in human endothelial cells was associated with a pro-inflammatory and endothelial-activated profile in immortalised HUVEC-derived cells, ECRF-24^6^ and knockdown of GLO1 in human aortic endothelial cells (HAECs) *in vitro* changed the expression of gene set linked to other coronary artery disease (CAD) associated genes in pathways of lipid metabolism, immune cell regulation, cell stress response, protein metabolism and DNA repair, presuming that GLO1 deficiency is involved in CAD development^19^.

This study aimed to investigate the impact of endogenous MG accumulation by GLO1-knockdown on primary HAECs in high glucose concentration on the cell proteome to identify possible MG-regulated target proteins. Furthermore, the effect of GLO1-knockdown on endothelial function estimates, oxidative stress, apoptosis and extracellular collagen content was analysed in this cell culture model.

## Materials and Methods

### Cell culture

Primary human aortic endothelial cells (HAECs, Lonza, Walkersville, USA, cat. No. CC-2535) were grown in insulin-free endothelial growth medium with 5 mM glucose supplemented with EGM-2 BulletKit (Lonza) at 37 °C in a humidified atmosphere of 5% CO_2_ to 70–80% confluence according to manufacturer’s instruction for primary cells to suppress differentiation and tube formation induced by confluent cultivation. Media conditions were adapted to hyperglycaemic conditions (25 mM glucose) if indicated 24 h after transfection; or at the appropriate time point for the hyperglycaemic control (named WT25mM).

### Gene specific knockdown

2 × 10^5^ cells were transfected with 5 nM siRNA GLO1-siRNA (Silencer Select Validated siRNA ID s5824, Ambion Applied Biosystems, USA: sense: 5′-GGCUUAUGAGGAUAAAAAUtt-3′, antisense 5′-AUUUUUAUCCUCAUAAGCCaa-3′) or with a non-target siRNA (NT-siRNA, Silencer Select Negative Control No. 1, Cat. No. 4390849, Ambion Applied Biosystems, USA) using Lipofectamine2000 transfection reagent (Invitrogen, Carlsbad, USA). 50 μM of siRNA and 3 μl of transfection reagent were diluted with Opti-MEM (Invitrogen), and incubated for 20 min at room temperature, and then added to the cells. 1.5 ml transfection medium (EGM-2 with 10% FBS, but without antibiotics) were added. 24 h after transfection, medium was replaced with fresh complete medium containing 25 mM glucose if indicated. Gene expression assays were performed 48 h after transfection and protein expression analysis was done 72 h after transfection, respectively.

### Chromatographic and mass spectrometric measurement of accumulated MG

MG was determined by derivatisation with 1,2-diaminobenzene and quantification of the quinoxaline adducts by stable isotopic dilution analysis liquid chromatography-tandem mass spectrometry (LC–MS/MS) as described elsewhere^19^,^20^. Briefly, 0.5–1 × 10^6^ cultured cells were sonicated and subsequently mixed with 4% ice-cold trichloroacetic acid-0.9% NaCl. Stable isotopic standard ([^13^C_3_]MG) was added and mixed, and samples were centrifuged. The supernatant was removed, sodium azide (0.3%) added and then finally 0.1 mM 1,2-diaminobenzene was added for derivatisation over 4 h. Calibration standards containing 2 pmol of isotopic standards and 0–20 pmol of MG were prepared and derivatised concurrently. Samples were analysed by LC–MS/MS with a C18 reverse-phase column (100 mm × 2.1 mm). The mobile phase was 0.1% trifluoroacetic acid in water with a linear gradient of 0–25% acetonitrile over 10 min. The quinoxaline adducts were detected by electrospray positive-ionisation multiple reaction monitoring.

### Measurement of protein glycation by MG

Protein glycation by MG was assessed by measurement of the major MG-derived AGE, MG-H1, determined investigator-blinded by LC-MS/MS with quantitation by stable isotopic dilution analysis from snap frozen HAEC cells or cell culture medium stored at −80 °C as previously described^21^. MG-H1 adduct residue content of cell protein was analysed after exhaustive enzymatic hydrolysis by consecutive incubation with pepsin, pronase E, and finally aminopeptidase and prolidase, under argon. MG-H1 free adduct concentration was also determined by direct analysis of ultrafiltrates (12 kDa cut-off) at baseline and at the end of the culture, thereafter deducing the flux of MG-H1 free adduct formation (pmol free adduct/day/10^6^ cells). MG-H1 was quantified by reference to calibration curve response of authentic standard. LC-MS/MS was performed with a Waters Acquity UPLC system with Quattro Premier XE tandem mass spectrometer.

### Cell protein analysis by two-dimensional gel electrophoresis with protein identification by proteomics mass spectrometry

#### Sample preparation

Cells were collected on ice, washed with ice-cold PBS and homogenised in lysis buffer (30 mM Tris, 2 M thiourea, 7 M urea, 4% 3-[C3-cholamidopropyl)dimethylammonio]-1-propanesulfonate (CHAPS, pH 8.5), and centrifuged at 16,000 × *g* for 15 min after sonication on ice. Supernatant was collected and protein concentration of each sample was determined using the Bradford assay (Bio-Rad, Hercules, USA). The protein samples were stored at −80 °C. Protein lysates of each sample (n = 6, each) were labelled with Cy3 or Cy5 ‘minimal dyes’; (GE Healthcare, Freiburg, Germany) whereas the Cy2 dye was exclusively used for labelling the internal standard representing a pool of all used samples. An aliquot of each sample (25 μg) was mixed with 200 pmol CyDye and kept on ice in the dark for 30 min. The labelling reaction was stopped by adding 5 pmol lysine to each sample, and incubated in the dark on ice for 10 min. The labelled samples were mixed at a ratio of 1:1:1 (Cy2:Cy3:Cy5).

#### Gel electrophoresis

Equal amounts of labelled proteins (75 μg) were separated by 2-dimensional gel electrophoresis following the method of Klose^20^. An internal standard, consisting of all samples, was used in all runs to control for gel-to-gel variation and facilitate image matching.

#### Image scan and data analysis

Gel images were scanned using a variable mode fluorescent scanner (Typhoon Trio, GE Healthcare) to generate gel images of Cy2, Cy3 and Cy5 labelled proteins. The scanned images were then analysed by the DeCyder-2D-V6.5 software package (GE Healthcare). Differential spots were defined by a p < 0.05 (unpaired t-test).

#### Protein identification by nanoflow mass spectrometry

Gels were collected manually and digested in-gel with trypsin (Promega, Mannheim, Germany). Tryptic peptides were extracted twice with 10 μl acetonitrile/formamide (5%) (50/50 [vol/vol]), extracts combined and solvent removed *in vacuo*. Residues were acidified by addition of 5% formamide (20 μl). Samples were analysed by nano-flow liquid chromatography electrospray ionisation-ion trap mass spectrometry (LC-MSn) using an Ultimate3000 HPLC system (Dionex LC Packings, Idstein, Germany) with an HCTultra PTM Discovery System ion trap mass spectrometer (Bruker Daltonics, Bremen, Germany) fitted with a nanoelectrospray ion source (Bruker Daltonics) and distal coated SilicaTips (FS360–20–10-D; New Objective, Woburn, MA, USA). Peptides were first separated by reverse phase nanoLC on a 75 μm (inner diameter) × 150 mm C18 PepMap column (Dionex LC Packings) with pre-concentration for 10 min with 0.1% trifluoroacetic acid on a μ-precolumn (300 μm (inner diameter) × 1 mm, C18 PepMap, Dionex LC Packings). For peptide separation a solvent system consisting of 0.1% (v/v) formamide (solvent A) and 0.1% (v/v) formamide, 84% (v/v) acetonitrile (solvent B) was used. The ion trap mass spectrometer was externally calibrated with standard compounds. Instrument settings were: capillary voltage 1400 V; endplate offset 500 V; dry gas flow 8.0 L/min; dry temperature, 160 °C; aimed ion charge control 150,000; maximal fill-time 500 ms. For mass spectrometry (MS) analyses, data-dependent software (HCT plus, Esquire Control V6.1, Bruker Daltonics) was employed. To generate fragment ions, low-energy collision-induced dissociation (CID) was performed on isolated multiply charged peptide ions with a fragmentation amplitude of 0.6 V. Exclusion limits were automatically placed on previously selected mass-to-charge ratios for 1.2 min. MS spectra were a sum of seven individual scans ranging from *m/z* 300–1,500 with a scanning speed of 8,100 (*m/z)*/s, while MS_n_ spectra (successive fragmentation of peptides) were a sum of four scans ranging from *m/z* 100–2,800 at a scan rate of 26,000 (*m/z)*/s. Peaklists of MS_n_ spectra were generated using the software tools DataAnalysis 4 with default parameters. For peptide and protein identification, peaklists were correlated with the human International Protein Index (Human IPI v.3.71 decoy database containing 86.745 protein and 86.745 decoy entries) using the ProteinScape (version 1.3, Bruker Daltonics, Bremen, Germany) database platform and MASCOT^21^ algorithm. All searches were performed with tryptic specificity allowing one missed cleavage and mass tolerances of 1.2 Da and 0.6 Da for MS and MS_n_ experiments, respectively. Cysteine modification with propionamide was considered as fixed and in-source oxidation of methionine as variable modification. Proteins were assembled on the basis of peptide identifications using the ProteinExtractor Tool (version 1.0) in ProteinScape and sorted according to their identification scores. Redundancies in protein entries are removed such that only the protein of lowest molecular mass is reported. Only proteins were considered as identified for which a minimum of 2 peptides had been found and which were no obvious contaminants (keratin 1, 2, 9 or 10). For the identification of single peptides minimal scores of 20 (MASCOT) was required. Protein names were assigned to individual spots according to highest MASCOT score.

### Immunoblot analysis

Cells were lysed in buffer containing NaCl (150 mM), Tris-HCl (50 mM), EDTA (1 mM), NaF (1 mM), Na_3_VO_4_ (1 mM), Nonidet P40 (1%), sodium deoxycholate (0.25%) and proteinase inhibitor cocktail (Sigma-Aldrich, St. Louis, USA). Proteins were separated by NuPAGE 4–12% Bis-Tris gel (Invitrogen) electrophoresis and electro transferred (Trans-blot SD, Bio-Rad, Hercules, USA) to nitrocellulose membranes (Bio-Rad). As molecular weight marker, Precision Plus Protein Standard (BioRad, Cat. No. 161-0374) was applied. After incubation for 1 h in blocking-solution containing 5% dry milk in Tris-buffered saline, membranes were washed in Tris-buffered saline and incubated overnight at 4 °C with the antibodies: polyclonal anti-glyoxalase 1 antibody (diluted 1:1000, Abcam, Cambridge, UK, cat. No. ab 96032), polyclonal anti-procollagen lysine oxoglutarate dehydrogenase 2 (PLOD2) antibody (diluted 1:1000, ProteinTech Group, Chicago, USA, cat. No. 21214-1-AP), polyclonal anti-prolyl 3-hydroxylase 3 (LEPREL2) antibody (diluted 1:800, ProteinTech Group, cat. No. 16023-1-AP), monoclonal anti-dihydropyrimidinase-related protein 2 (DPYSL2) antibody (diluted 1:1000, LifeSpan BioSciences Inc., Seattle, WA, cat. No. LS-B3616), polyclonal anti-Caspase-3 antibody (diluted 1:1000, Cell Signaling Technology Inc. MA, USA, cat. No. 9662), polyclonal anti-Col1A antibody (diluted 1:1000, LifeSpan Biosciences Inc. Seattle, USA, cat. No. LS-C-150353), monoclonal anti-Col5A1 antibody (diluted 1:200, Acris Antibodies GmbH, Herford Germany cat. No. AF6110), monoclonal anti-total eNOS antibody (diluted 1:4000, Abcam, cat-No. ab76198), polyclonal anti-eNOSphosphoS1177 antibody (diluted 1:2000, Abcam, cat. No. ab184154), polyclonal anti-eNOSphosphoS615 antibody (diluted 1:2000, Abcam, cat. No. ab138458), polyclonal anti-eNOSphosphoT494 antibody (diluted 1:2000, Abcam, cat. No. ab138430), and polyclonal anti-PARP antibody, (diluted 1:1000, Cell Signaling Technology, cat. No. 9542). Polyclonal anti-β-actin antibody (diluted 1:1000, Cell Signaling Technology, cat. No. 3700) or monoclonal anti-alpha-tubulin (diluted 1:4000, Cell Signaling Technology, cat. No. 2125) were used to detect the reference protein (loading control). For further information on the antibodies used, see Supplemental information. After washing, blots were incubated for 1 h with horseradish peroxidase (HRP)-conjugated secondary antibodies at room temperature. As a HRP substrate ChemiGlow (Alpha Innotec Corporation, San Leandro, USA) was used and recorded by a CCD-camera system (Fluor Chem FC3, CellBiosciences, Santa Clara, USA). Wherever possible, quantitative comparisons between samples have been done on the same blot, in cases where a comparison was not possible on one blot, different blots were used, with samples derived from the same experiment, analysed in the same concentration and in parallel.

### ELISA

The vimentin concentration was determined by performing ELISA (USCN Life Science Inc, Wuhan, China) according to the manufacturer’s instructions using 5 μg/mL protein from whole cell lysate. Measurements on the expression of MCP-1 (R&D Systems™, Minneapolis, U.S.A.) was done in cell culture supernatants in a 1:5 dilution with PBS, endothelin-1 (R&D Systems™) in a 1:50 dilution, sICAM-1 (Abcam), and sVCAM-1 (Abcam) were done in cell culture supernatants according to the manufacturer’s instructions without further dilution.

### Measurement Caspase-3/7-activity

HAECs were seeded at 12.500 cells per well and transfected with NT-siRNA or GLO1-siRNA. Caspase-3/-7 activity was measured using the Caspase-Glo 3/7-Assay (Promega, Madison, WI, USA) according to manufacturer’s instructions. The assay monitors the Caspase-3/7 cleavage of the luminogenic substrate containing the DEVD sequence. Following caspase cleavage, a substrate for luciferase (aminoluciferin) is released, resulting in the luciferase reaction which is analysed. Caspase activity is given as relative light units (RLUs).

### Measurement of apoptosis by flow cytometry

HAECs transfected with NT-siRNA or GLO1-siRNA were double-stained with annexin V–FITC and propidium iodide (PI) (Beckman Coulter, Krefeld, Germany), according to the manufacturer’s instructions, and immediately analysed by flow cytometry (FACScan, Becton Dickinson, Franklin Lakes, USA). Proportion of apoptotic cells was calculated as percentage of the number of annexin V positive cells.

### Measurement of ROS generation by flow cytometry

SiRNA-transfected cells were washed twice with Hank’s buffered saline solution (HBSS, pH 7.4) (Sigma-Aldrich, St. Louis, USA). Afterwards, 2′7′-dichlorodihydrofluorescein diacetate (DCF-DA) (Sigma-Aldrich St. Louis, USA) was added followed by incubation at 37 °C for 30 min in the dark. Cells were then collected with trypsin, suspended in HBSS buffer, centrifuged (5 min; 400 × *g*) and suspended again in 0.5 ml HBSS buffer. Fluorescence intensity of 5,000 cells was measured by flow cytometry (FACScan, Becton Dickinson, Franklin Lakes, USA).

### Measurement of GLO1 activity

The activity of GLO1 was measured by determining the isomerisation rate of hemithioacetal, formed non-enzymatically by pre-incubation of MG and reduced glutathione (GSH), to S-D-lactoylglutathione, followed spectrophotometrically at 240 nm; Δε_240_ = 2.86 mM^−1^cm^−1^ 22. GLO1 activity is given in U per mg protein. Blank correction for absorbance at 240 nM was performed.

### Isolation of total RNA and real time PCR

Isolation of total RNA was performed using the RNeasy Kit (Qiagen, Hilden). Reverse transcription of cellular RNA used 250 ng of total RNA and 100 U of superscript II (Invitrogen). Real-time PCR was performed using MasterPlex (Eppendorf, Hamburg) and Platinum SYBR-Green qPCR SuperMix-UDG (Invitrogen) for detection using appropriate oligonucleotides and amplification conditions. The RNA content was normalised to the reference genes RPL32, β-actin or β-microglobulin either alone or in a combined fashion. Oligonucleotide sequences are available from the author upon request.

### Statistics

Results of the experimental studies are reported as mean±SEM, unless otherwise stated. Differences were analysed by one-way ANOVA after checking for normality via Bartlett’s test followed by Tukey’s multiple comparison post-test using GraphPad Prism version 6.05 for Windows (GraphPad Software, San Diego, CA, USA). P values p < 0.05 were regarded statistically significant. In the following N describes independent biological experiments, whereas n accounts for the number of repetitions of measurements.

## Results

### GLO1 expression and activity of HAECs incubated in low and high glucose conditions and effect of GLO1-knockdown

**Figure 2:**
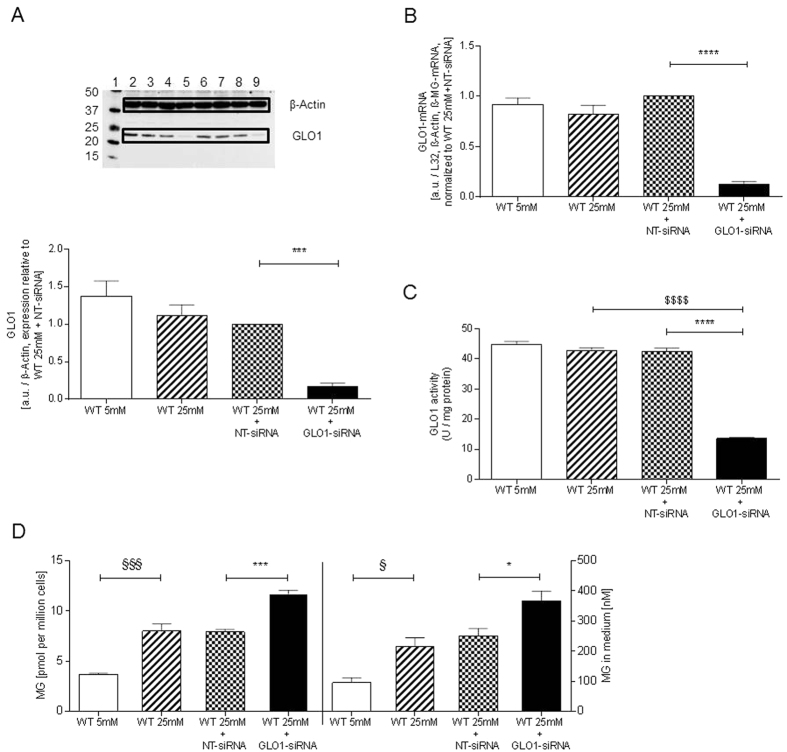
GLO1 Expression and MG formation. (**A**) Western Blot analysis for GLO1 of HAECs cultured under normo- and hyperglycaemic conditions and after transfection with non-target (WT25mM+NT-siRNA) and GLO1 specific siRNA (WT25mM+GLO1-siRNA) (following 72 h siRNA transfection). (N = 5). lane 1: Precision Plus Protein Standard; lane 2, 6: WT5mM; lane 3, 7: WT25mM, lane 4, 8: WT25mM+NT-siRNA; lane 5, 9: WT25mM+GLO1-siRNA. ****p < 0.0001 vs. WT25mM+NT-siRNA. (**B**) mRNA Expression of GLO1 in HAECs cultured under normo- and hyperglycaemic conditions and after transfection with NT- and GLO1-siRNA (following 48 h siRNA transfection). (N = 5, n = 2). ****p < 0.0001 vs. WT25mM+NT-siRNA. (**C**) GLO1 activity for WT5mM, WT25mM, WT25mM+NT-siRNA, and WT25mM+GLO1-siRNA. (N = 3, n = 3). *p < 0.05 vs. WT25mM+NT-siRNA, ^$^p < 0.05 vs. WT25mM. (N = 3, n = 3). (**D**) MG content of HAECs (left diagram) and MG concentration of culture medium (right diagram) for WT5mM, WT25mM, WT25mM+NT-siRNA, and WT25mM+GLO1-siRNA. Significance: ^§^p < 0.05, ^§§§^p < 0.001 vs. WT5mM, *p < 0.05, ***p < 0.001 vs. WT25mM+NT-siRNA. (N = 3, n = 3).

HAECs were cultured and transfected as described above. GLO1 protein and mRNA was quantified in HAECs cultured with 5 mM glucose (WT5mM). Both mRNA and protein levels were neither changed significantly by culture with 25 mM glucose (WT25mM) nor by culture with high glucose concentration with NT-siRNA (WT25mM+NT-siRNA) within the chosen observation period. Incubation with 25 mM glucose and GLO1-siRNA (WT25mM+GLO1-siRNA) decreased both GLO1 mRNA and protein by 87%, compared to the WT25mM+NT-siRNA, and by 90% compared to WT5mM 72 h after transfection (Fig. 2A,B). This knockdown was confirmed by the use of three different siRNAs. For the following experiments a high validated selected siRNA was used. GLO1 activity of HAECs cultured with low glucose concentration (WT5mM) was 44.88 ± 0.97 U/mg protein (N = 3, n = 3). It was unchanged in WT25mM (42.64 ± 1.08 U/mg protein) and WT25mM+NT-siRNA (42.50 ± 1.11 U/mg protein) but decreased 68% in WT25mM+GLO1-siRNA (13.65 ± 0.32 U/mg protein, p < 0.0001 vs. WT25mM+NT-siRNA) (Fig. 2C). A follow up of 7 days revealed that GLO1 knockdown was stable for at least four days, presenting with the lowest level of GLO1 after 3 days, which corresponded to mRNA data proving a stable knockdown on mRNA level for 72 h. For this reason, a culture period of 72 h was chosen for protein analyses for the following experiments.

The MG content of HAECs was 3.71 ± 0.12 pmol/10^6^ cells in WT5mM. MG content increased approx. 2.2fold in WT25mM and WT25mM+NT-siRNA but increased approx. 3.2fold in WT25mM+GLO1-siRNA (Fig. 2D). The MG concentration in the culture medium similarly followed this trend. In WT5mM the MG concentration was 96 ± 17 nM and increased approx. 2.3fold in WT25mM or WT25mM+NT-siRNA but increased approx. 3.8fold in WT25mM+GLO1-siRNA (Fig. 2D).

### Increased MG-derived AGE with GLO1-knockdown

We quantified MG-H1 residue content of cell protein and flux of MG-H1 free adduct accumulation in the culture medium – the latter originating mainly from proteolysis of MG-H1 containing cell proteins with a minor contribution from direct reaction of MG with arginine in the culture medium. MG-H1 residue content of cell protein was 0.993 ± 0.088 mmol/mol arg in control cultures and surprisingly was not increased in hyperglycaemic cultures with or without NT and GLO1-siRNA. The flux of MG-H1 free adduct was, however, increased *approx.* 2fold in hyperglycaemic cultures and in cultures with high glucose concentration and NT-siRNA. It was further increased, *approx.* 8fold with high glucose concentration and GLO1-siRNA (Table 1). This suggests an increased glycation of proteins by MG in hyperglycaemic cultures and an exacerbation by knockdown of GLO1 but over the relatively short period studied (48 h) proteins modified were efficiently targeted to proteolysis and the MG-H1 adducts are excreted (Table 1).

### GLO1-knockdown in HAECs resulted in changed abundance of mainly structural proteins and collagen modifying proteins

HAECs were cultured as described above and afterwards analysed for changes in the cellular proteome. Cell protein extracts were separated by 2D-DIGE. In HAECs with GLO1-knockdown there were 9 proteins decreased significantly with a minimum of - 1.4 fold-change, p < 0.05 (Table 2).

**Figure 3:**
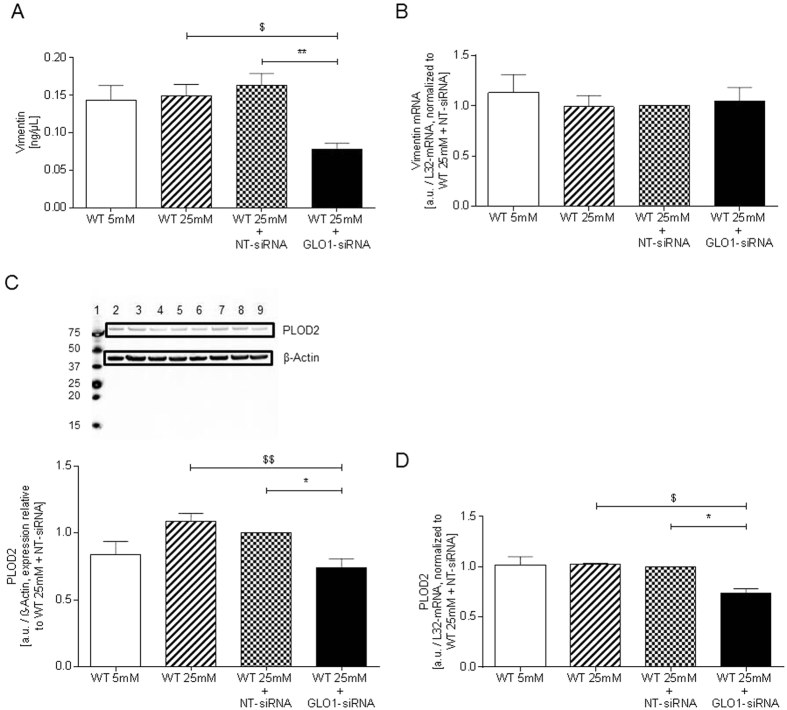
Vimentin and PLOD2. (**A**) Vimentin concentration determined by ELISA (N = 4, n = 3). **p < 0.01 vs. WT25mM+NT-siRNA. ^$^p < 0.05 vs. WT25mM. (**B**) mRNA Expression of vimentin in HAECs transfected with scrambled and GLO1-siRNA following 72 h siRNA transfection (N = 3, n = 2). (**C**) Western blot analysis performed with anti-PLOD2 in lysates from siRNA transfected HAEC (N = 4, n = 2). lane 1: Precision Plus Protein Standard; lane 2, 7: WT25mM; lane 3, 8: WT25mM+NT-siRNA; lane 4, 9: WT25mM+GLO1-siRNA; lane 5, 6: WT5mM,: *p < 0.05 vs. WT25mM+NT-siRNA. ^$^p < 0.05 vs. WT25mM. (**D**) mRNA Expression of PLOD2 in HAECs transfected with NT- and GLO1-siRNA (following 72 h siRNA transfection). (N = 4, n = 2). *p < 0.05 vs. WT25mM+NT-siRNA. ^$^p < 0.05 vs. WT25mM.

Two main classes of proteins were identified: structural proteins, such as vimentin, α-tubulin 1B and lamin A; and second enzymes that modify collagen, such as procollagen lysine oxoglutarate dehydrogenase 2 (PLOD2) that hydroxylates lysine at the 5-position in collagens, and prolyl 3-hydroxylase 3 (LEPREL2) that hydroxylates proline at the 3-position particularly in collagen-4 and collagen-5. Decrease of lamin A and tubulin could not be verified by Western blotting, likely due to low protein concentrations of the identified protein isoforms. Vimentin was the most changed protein, the abundance decreased 50%, which was confirmed by ELISA: vimentin (ng/μl), WT25mM+NT-siRNA 0.17 ± 0.02 vs. WT25mM+GLO1-siRNA 0.08 ± 0.01, p < 0.01 (Fig. 3A). A similar decrease in vimentin mRNA was not observed (Fig. 3B).

Down-regulation of PLOD2 in GLO1-knockdown in HAECs observed by 2D-DIGE was corroborated by Western blotting. PLOD2 protein was decreased by GLO1-knockdown compared to WT25mM+NT-siRNA (0.10 ± 0.01 vs. 0.14 ± 0.01 a.u., p < 0.01 vs. WT25mM+NT-siRNA, normalised to β-actin). PLOD2 mRNA was also decreased: 0.84 ± 0.08 vs. 1.14 ± 0.06 a.u., p < 0.05 vs. WT25mM+NT-siRNA (Fig. 3C,D).

**Figure 4:**
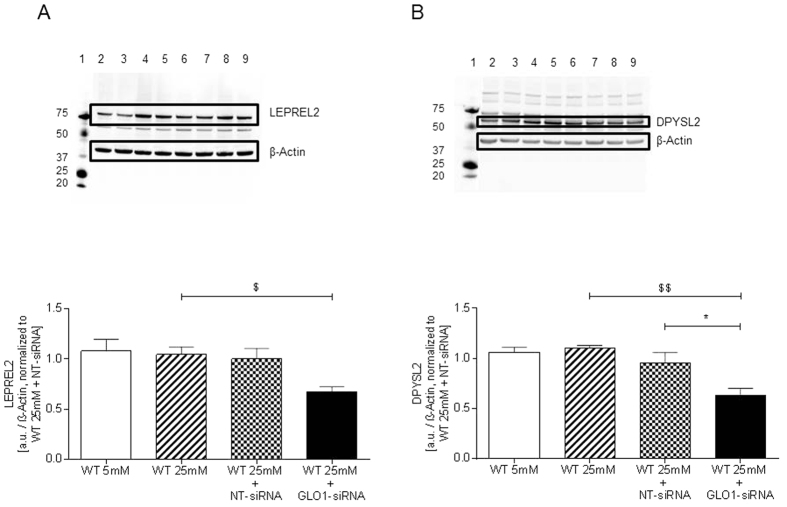
LEPREL2 and DYSPL2. (**A**) Western blot analysis performed with anti-LEPREL2 in lysates from siRNA transfected HAEC (N = 3, n = 3). ^$^p < 0.05 vs. WT25mM. lane 1: Precision Plus Protein Standard; lane 2, 3: WT25mM+GLO1-siRNA; lane 4, 5: WT25mM+NT-siRNA, lane 6, 7: WT25mM; lane 8, 9: WT5mM. (**B**) Western blot analysis performed with anti-DPYSL2 in lysates from siRNA transfected HAEC (N = 4, n = 2). *p < 0.05 vs. WT25mM+NT-siRNA. ^$$^p < 0.05 vs. WT25mM. lane 1: Precision Plus Protein Standard; lane 2, 3: WT25mM+GLO1-siRNA; lane 4, 5: WT25mM+NT-siRNA, lane 6, 7: WT25mM; lane 8, 9: WT5mM.

The decrease of LEPREL2 and dihydropyrimidinase-related protein 2 (DPYSL2) protein observed by 2D-DIGE in GLO1-knockdown in HAECs was confirmed by Western blotting (0.67 ± 0.05 vs. 1.00 ± 0.11 a.u., p < 0.05 vs. WT25mM and 0.63 ± 0.07 vs. 0.96 ± 0.10 a.u., p < 0.05 vs. WT25mM+NT-siRNA, respectively) (Fig. 4A,B).

### Effect of MG accumulation of apoptosis

Increased apoptosis and reduced viability was detected following GLO1-knockdown as suggested by proteome analysis as non-significant up regulation (FC 1.2 vs. WT25mM+NT-siRNA) of Annexin-A1 expression by increase in Annexin-V-FITC binding on the cell surface.

**Figure 5:**
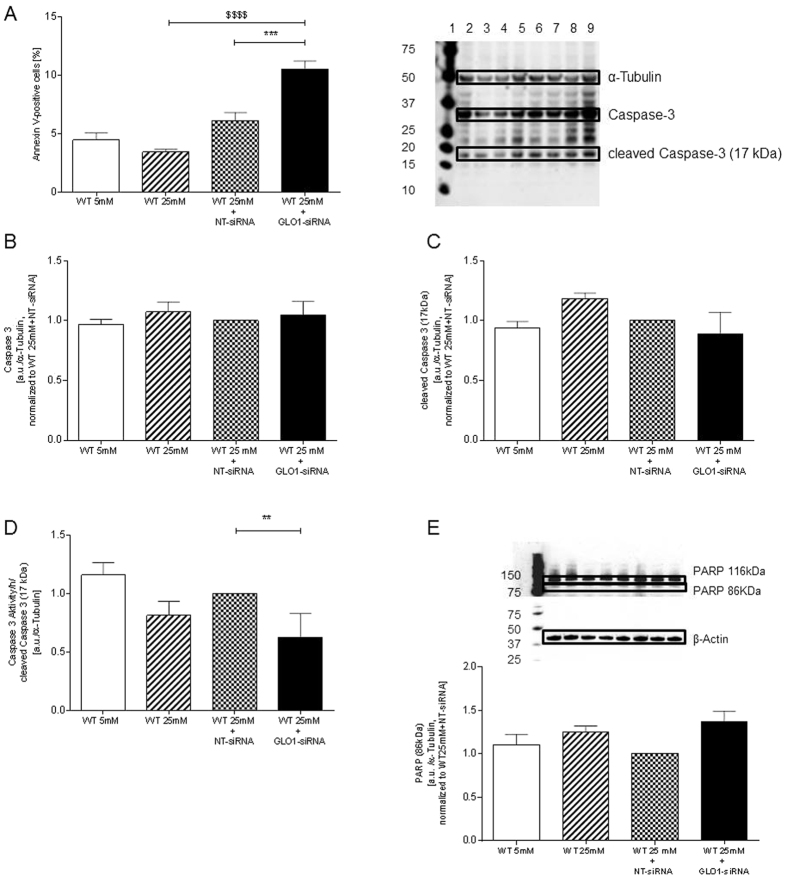
Apoptosis, expression and activity of caspase 3. (**A**) Ratio of apoptotic cells cultured under normo- and hyperglycaemic conditions and after transfection with non-target (WT25mM+NT-siRNA) and GLO1 specific siRNA (WT25mM+GLO1-siRNA) (following 72 h siRNA transfection). (N = 6, n = 2). ***p < 0.001 vs. WT25mM+NT-siRNA. ^$$$$^p < 0.0001 vs. WT25mM. (**B**) Western Blot analysis for Caspase 3 of HAECs cultured under normo- and hyperglycaemic conditions and after transfection with non-target (WT25mM+NT-siRNA) and GLO1 specific siRNA (WT25mM+GLO1-siRNA) (following 72 h siRNA transfection). (N = 5). lane 1: Precision Plus Protein Standard; lane 2, 6: WT25mM; lane 3, 7: WT5mM, lane 4, 8: WT25mM+NT-siRNA; lane 5, 9: WT25mM+GLO1-siRNA. (**C**) Western Blot analysis for cleaved Caspase 3 (17 kDa) of HAECs cultured under normo- and hyperglycaemic conditions and after transfection with non-target (WT25mM+NT-siRNA) and GLO1 specific siRNA (WT25mM+GLO1-siRNA) (following 72 h siRNA transfection). (N = 5). lane 1: Precision Plus Protein Standard; lane 2, 6: WT25mM; lane 3, 7: WT5mM, lane 4, 8: WT25mM+NT-siRNA; lane 5, 9: WT25mM+GLO1-siRNA. (**D**) Caspase 3/7 activity of HAECs cultured under normo- and hyperglycaemic conditions and after transfection with non-target (WT25mM+NT-siRNA) and GLO1 specific siRNA (WT25mM+GLO1-siRNA) (following 72 h siRNA transfection). (N = 3, n = 3), **p < 0.01 vs. WT25mM+NT-siRNA. (**E**) Western Blot analysis for PARP (86 kDa) of HAECs cultured under normo- and hyperglycaemic conditions and after transfection with non-target (WT25mM+NT-siRNA) and GLO1 specific siRNA (WT25mM+GLO1-siRNA) (following 72 h siRNA transfection). (N = 5). lane 1: Precision Plus Protein Standard; lane 2, 6: WT5mM; lane 3, 7: WT25mM, lane 4, 8: WT25mM+NT-siRNA; lane 5, 9: WT25mM+GLO1-siRNA.

Incubation of HAECs with 25 mM glucose for 48 h did not significantly increase Annexin-V-FITC binding on the cell surface (4.5 ± 0.6% vs. 3.4 ± 0.2%, WT25mM vs. WT5mM; 6.1 ± 0.7% vs. 3.4 ± 0.2%, WT25mM+NT-siRNA vs. WT5mM)). However, incubation of HAECs with 25 mM glucose for 48 h and with GLO1-knockdown induced apoptosis, as judged by binding of Annexin-V-FITC: 10.5 ± 0.7% vs. 6.1 ± 0.7%, p < 0.001 vs. WT25mM+NT-siRNA; p < 0.0001 vs. WT25mM (Fig. 5A).

To further analyse the involved pathways expression levels of procaspase 3 and cleaved caspase 3 as well as caspase 3/7 activity and PARP were analysed. Expression of procaspase 3 and cleavage of caspase 3 were unchanged following knock down of GLO1 (Fig. 5B,C). Caspase 3/7 activity was significantly downregulated in WT25mM-GLO1-siRNA by 37% whereas PARP cleavage fragment (86 kDa) was slightly, but not significantly increased under these conditions (Fig. 5D,E).

### GLO1-knockdown in HAECs increased inflammation and markers of endothelial dysfunction

**Figure 6:**
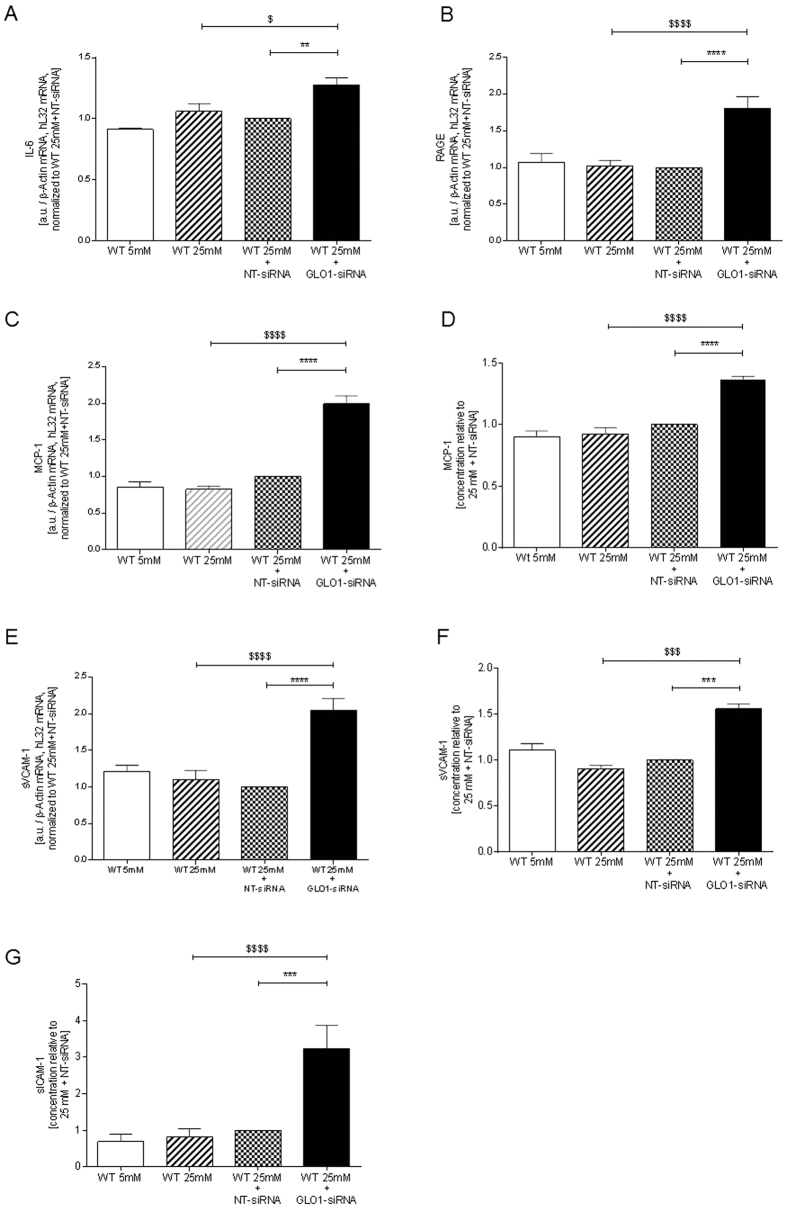
Inflammation and endothelial dysfunction. Expression of genes involved in inflammation and endothelial function (IL-6 (**A**), RAGE (**B**), MCP-1(**C**), sVCAM-1 (**E**)) were analysed on mRNA level (N = 3, n = 3). **p < 0.01, ****p < 0.0001 vs. WT25mM+NT-siRNA, respectively. ^$^p < 0.05, ^$$$$^p < 0.0001 vs. WT25mM. MCP-1 (**D**), sVCAM-1 (**F**), and sICAM-1 (**G**) were further analysed on protein level in the cell supernatant fractions via ELISA (N = 3, n = 3). ***p < 0.001, ****p < 0.0001 vs. WT25mM+NT-siRNA, ^$$$^p < 0.001, ^$$$$^p < 0.0001 vs. WT25mM.

To assess whether intracellular accumulation of MG by GLO1-knockdown induced endothelial dysfunction and inflammation, expression of IL-6, RAGE, MCP-1 and soluble ICAM-1 and VCAM-1 were analysed. Compared to WT25mM+NTsiRNA knockdown of GLO1 induced significantly increased IL-6 (1.28 ± 0.06 fold), RAGE (1.81 ± 0.16 fold), and MCP-1 (1.99 ± 0.11 fold) mRNA (Fig. 6A–C). In conditioned medium of HAEC cultures, siRNA-mediated knockdown of GLO1 significantly increased protein levels of MCP-1 (1.37 ± 0.02 fold), sVCAM-1 (1.56 ± 0.05 fold), and sICAM-1 (3.24 ± 0.63 fold) (Fig. 6D,F,G) if compared to WT25mM+NT-siRNA. In addition, mRNA levels of VCAM-1 were found to be elevated (2.05 ± 0.16 fold) following GLO1-siRNA mediated knockdown in comparison to WT25mM+NT-siRNA (Fig. 6E).

### GLO1-knockdown in HAECs increased markers of vasoconstriction without affecting vasorelaxation markers

**Figure 7:**
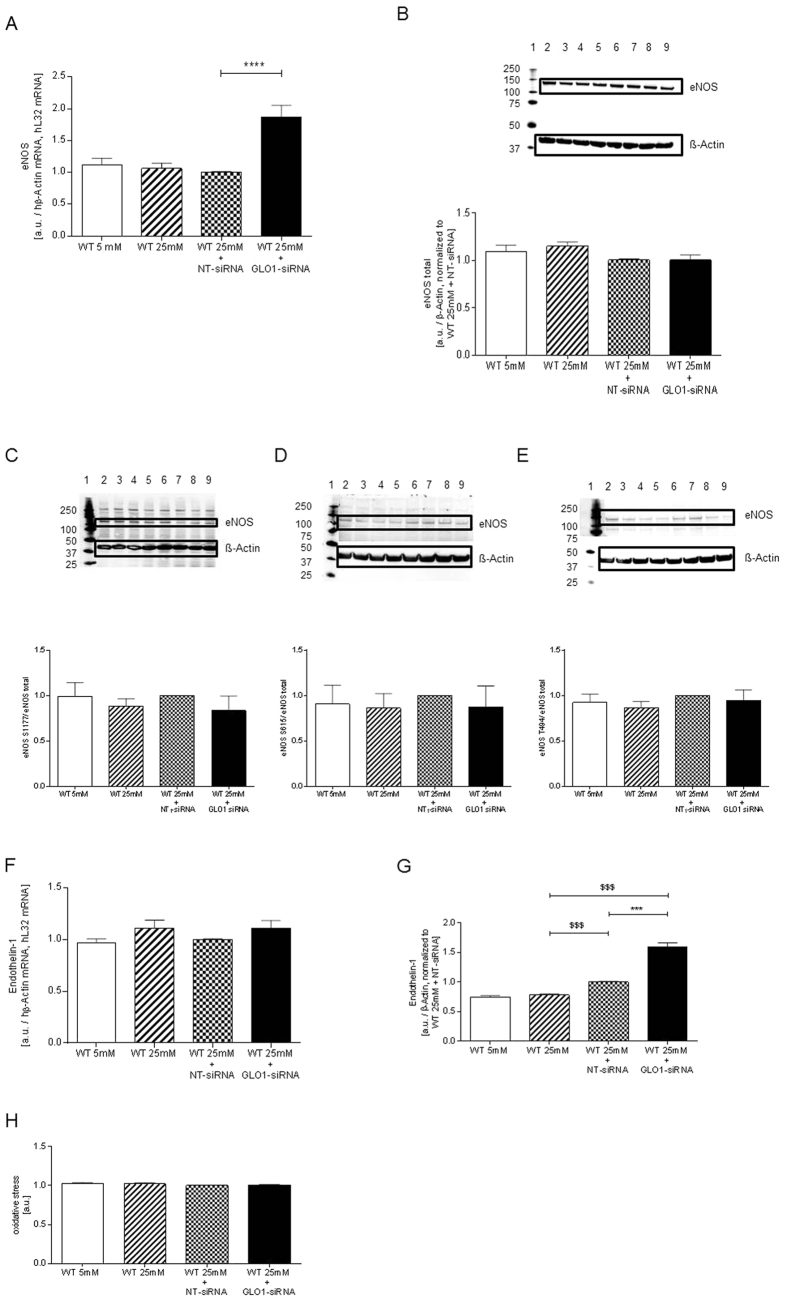
Expression and phosphorylation of eNOS, expression of endothelin-1, and oxidative stress. (**A**) mRNA expression of eNOS of HAECs cultured under normo- and hyperglycaemic conditions and after transfection with non-target (WT25mM+NT-siRNA) and GLO1 specific siRNA (WT25mM+GLO1-siRNA) (following 48 h siRNA transfection). (**B**–**E**) Western Blot analysis for eNOS (**B**), eNOS phosphorylation of Ser1177 (**C**), Ser615 (**D**), and Thr494 (**E**) of HAECs cultured under normo- and hyperglycaemic conditions and after transfection with non-target (WT25mM+NT-siRNA) and GLO1 specific siRNA (WT25mM+GLO1-siRNA) (following 72 h siRNA transfection). (N = 5). Different blots were used, with samples derived from the same experiment, analysed in the same concentration and in parallel to calculate the rate of phosphorylation of eNOS after determination of the concentration using β-actin as control on the same individual blot. lane 1: Precision Plus Protein Standard; lane 2, 6: WT25mM+GLO1-siRNA; lane 3, 7: WT25mM+NT-siRNA, lane 4, 8: WT25mM; lane 5, 9: WT5mM. (**F**) mRNA expression of endothelin-1 of HAECs cultured under normo- and hyperglycaemic conditions and after transfection with non-target (WT25mM+NT-siRNA) and GLO1 specific siRNA (WT25mM+GLO1-siRNA) (following 48 h siRNA transfection). (**G**) Endothelin-1 level were analysed extracellularly by ELISA. (N = 3, n = 3). ***p<0.001 vs. WT25mM+NT-siRNA, respectively. ^$$$^p < 0.001 vs. WT25mM. (**H**) Mean fluorescence intensity of HAECs cultured under normo- and hyperglycaemic conditions and after transfection with non-target (WT25mM+NT-siRNA) and GLO1 specific siRNA (WT25mM+GLO1-siRNA) (following 72 h siRNA transfection). (N = 3, n = 3).

To assess whether intracellular accumulation of MG by GLO1-knockdown induced endothelial dysfunction via the vasorelaxation/-constriction balance, expression levels of eNOS were analysed via Western Blot and expression of endothelin-1 was assessed by ELISA. While mRNA levels of eNOS were significantly upregulated (Fig. 7A), neither the protein levels of eNOS were influenced (1.05 ± 0.10 vs. 1.22 ± 0.05 a.u., p = ns vs. WT25mM and 1.05 ± 0.10 vs. 1.02 ± 0.02 a.u., p = ns vs. WT25mM+NT-siRNA, respectively, Fig. 7B) nor the eNOS-phosphorylation status (S1177, S651 and T494) (Fig. 7C–E). The expression of endothelin-1 was increased following unspecific knockdown and even more after GLO1-knockdown (1.00 ± 0.01 vs. 0.78 ± 0.02 a.u., p < 0.001 vs. WT25mM and 1.60 ± 0.06 vs. 1.00 ± 0.01 a.u., p < 0.001 vs. WT25mM+NT-siRNA, respectively) (Fig. 7F–G). In this context oxidative stress was determined by flow cytometry and was found to be not elevated following hyperglycaemic stress or GLO1-knockdown (Fig. 7H).

### GLO1-knockdown in HAECs increased extracellular concentrations of Collagen-1 and Collagen-5

**Figure 8:**
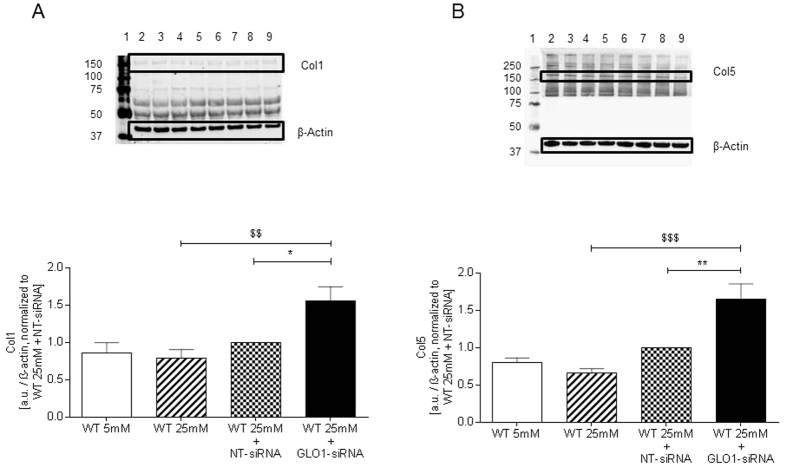
Collagen expression. Western Blot analysis for expression of Collagen-1 (**A**), and Collagen-5 (**B**), in cell culture supernatants of HAECs cultured under normo- and hyperglycaemic conditions and after transfection with non-target (WT25mM+NT-siRNA) and GLO1 specific siRNA (WT25mM+GLO1-siRNA) (following 72 h siRNA transfection). (N = 3, n = 3). lane 1: Precision Plus Protein Standard; lane 2, 6: WT25mM+GLO1-siRNA; lane 3, 7: WT25mM+NT-siRNA, lane 4, 8: WT25mM; lane 5, 9: WT5mM. **p < 0.01 vs. WT25mM+NT-siRNA, ^$$$^p < 0.001, ^$$$$^p < 0.0001 vs. WT25mM, respectively.

To assess whether intracellular accumulation of MG by GLO1-knockdown induced changes in the extracellular levels of collagen-1 and collagen-5 cell culture supernatants were analysed accordingly. Both, collagen-1 (1.38 ± 0.09 vs. 0.71 ± 0.07 a.u., p < 0.0001 vs. WT25mM and 1.38 ± 0.09 vs. 0.90 ± 0.11 a.u., p < 0.01 vs. WT25mM+NT-siRNA, respectively) as well as collagen-5 levels (1.61 ± 0.15 vs. 0.82 ± 0.14 a.u., p < 0.0001 vs. WT25mM and 1.61 ± 0.15 vs. 1.00 ± 0.07 a.u., p < 0.01 vs. WT25mM+NT-siRNA, respectively) were significantly upregulated (Fig. 8A,B).

## Discussion

To develop further the link of increased MG levels in HAECs and vascular dysfunction, we investigated the effects of GLO1-knockdown in HAECs in high glucose concentration as a model of hyperglycaemia. As the cells were grown in normoglycaemic media before we provoked the maximum phenotype by switching to hyperglycaemia and GLO1-knockdown simultaneously. Hyperglycaemia per se for the duration of 3 days did not induce the hyperglycaemic phenotype with the effects on GLO1 expression and activity shown by others in long-term culture experiments. For example, the downregulation of the activity of GLO1 in endothelial cells was detected in long-term culture under hyperglycaemic conditions which is more than double of our incubation period. Given the long half-life of GLO1 (179 h) remarkable effects are seen after a 1-week incubation period, which we have evaluated in previous experiments.

We chose primary human aortic endothelial cells to study for relevance to CVD. A recent clinical study of >90,000 CVD cases and controls linked GLO1 to CVD risk^19^. Increased exposure of HAEC to MG, as found in diabetes, and related dysfunction may contribute to this association and direct future research for improved prevention and treatment of CVD. Knockdown of GLO1 in HAECs influenced the expression of many genes that were also affected by CAD-associated SNPs in human genome wide association studies. The authors assume that the glyoxalase system may represent one common pathway responsible for both the microvascular and macrovascular complications observed in subjects with diabetes^19^.

Knockdown of GLO1 exacerbated MG accumulation in HAECs in high glucose concentration, increasing from 2–3 fold increase of MG content of cells. The increase in MG found in our study was of similar level achieved with the GLO1 inhibitor BBGD that increased EC anoikis^15^ and was prevented by overexpression of GLO1^16^. Similar proportionate increases in the MG concentration of culture medium are expected as MG permeates cell membranes with a half-life of <15 min^23^. The cell and medium concentration of MG increased in high glucose concentration mainly due to increased MG formation; metabolism by GLO1 activity was not decreased significantly. Longer incubations of HAECs in high glucose concentration, however, led to decreased GLO1 activity and protein^14^; comparable to our data of short term incubation with GLO1 knockdown. Increase of ROS production also was a characteristic of longer term incubation of HAECs with high glucose concentration than the 48 h period studies herein^14^. We were able to show that the observed effects are not related to the generation of oxidative stress, but obviously directly to MG accumulation. We analysed mRNA levels 48 h after transfection, whereas protein levels were determined after 72 h. This was done to harmonize protein levels to mRNA levels and to overcome time dependent shifts.

The cellular concentration of MG does not increase proportionately to the decrease in GLO1 activity because residual enzyme activity metabolizes MG and, with accumulation, more MG is consumed by glycation of protein and DNA^24^. The increased formation of MG glycated proteins is expected to lead to decreased half-life and decreased steady-state concentration of these proteins unless there is compensatory increased gene expression.

Vimentin, PLOD2, DPYSL2 and LEPREL2 proteins have decreased abundance in HAECs with knockdown of GLO1 and incubation with 48 h in high glucose concentration. Vimentin is a type-3 intermediate filament protein, part of the cytoskeleton of mesenchymal cells and thus contributes to endothelial cellular stability and integrity^25^. Vimentin had decreased protein but not mRNA with GLO1 silencing. The half-life of vimentin mRNA is *approx*. 6 h and the half-life of insoluble vimentin, the majority of vimentin, is long-lived^26^,^27^. This suggests that exposure to MG likely decreased translation where a decrease in short-lived vimentin mRNA is expected but a change in long-lived vimentin protein is not. Vimentin is susceptible to MG modification although the effect of this on vimentin protein half-life has not been studied^28^. In human dermal fibroblasts MG led to intracellular rearrangement and aggregation of vimentin^28^. MG-modified vimentin is likely to be in the long-lived insoluble fraction which was not studied herein. Decreased vimentin may contribute to increased apoptosis detected in GLO1-knockdown HAECs. This also likely compromises the vascular permeability barrier^29^ and results in disruption of the endothelial monolayer or shedding of endothelial cells and pronounced arterial stiffness^30^. Other contributions to HAEC loss *in vivo* likely come from MG-stimulated detachment from collagen-IV of the basement membrane and anoikis^15^; increased circulating endothelial cell number is characteristic for patients with type 2 diabetes and is a risk predictor for CVD^30^.

Collagen modification and modulation of its expression by MG is of importance in aging and diabetes. As major components of the arterial wall collagen provides the structural framework while elastin provides extensibility, contributing only to a minor extend to the mechanical and stability of the vessel. However, collagen does have limited elastic properties. With aging and diabetes elastin is replaced by collagen, leading to arterial stiffness and endothelial dysfunction^31^.

PLOD2 mRNA and protein has half-lives of ca. 9 h and 35 h, respectively^32^ and so may respond within the incubation period of 48 h if expression is decreased. Indeed, exposure to increased MG decreased both PLOD2 mRNA and protein, consistent with MG driving a decrease at the transcriptional level. PLOD2 belongs to a family of posttranslational modifying enzymes of collagen that provide a crucial hydroxylation step in collagen biosynthesis and is important for the correct folding and structure of collagen^33^,^34^,^35^,^36^,^37^. Different studies have shown that hypertension and diabetes promote vascular changes and instability of collagen^38^,^39^,^40^,^41^. Reduced availability of PLOD2 results in impaired collagen tensile strength and may account for partial loss of endothelial integrity. The mechanism by which MG reduces PLOD2 transcription may be linked to hypoxia-inducible factor-1 alpha that is known to positively induce PLOD2 expression in vascular endothelial cells^42^. HIF-1alpha protein levels and translational activity is compromised by methylglyoxal through different mechanisms which are elegantly described elsewhere^43^.

DPYSL2 is involved in the assembly of microtubules mainly in axonal growth but its role in endothelial function is yet unknown^44^. DPYSL2 and LEPREL2 mRNA and protein have half-lives of ca. 6 h and 106 h, and ca. 16 h and 78 h, respectively^32^, and hence the decrease in DPYSL2 and LEPREL2 protein only is likely due to increased degradation of these proteins, probably after MG modification. Reduction of PLOD2 and LEPREL2 by MG may synergize to provide abnormal post-translational processing of collagen and instability by abnormal lysyl-crosslinking and proline-3-hydroxylation.

The flux of MG-H1 free adducts was significantly increased in HAECs with incubation and high glucose concentration and GLO1-knockdown. This is consistent with increased glycation of cellular protein arising from increased MG and also glyoxal. The lack of increase in MG-H1 residue content of cell protein indicates that the MG-modified proteins are effectively proteolysed and the AGE adducts excreted. This will, however, decrease the half-life of modified proteins which will either decrease the cell content of unmodified proteins – such as we have detected - or induce compensatory increased protein expression and thereby add additional stress to maintain proteome homeostasis.

Exposure of HAECs to increased MG by knockdown of GLO1 increased the expression of inflammatory markers, IL-6, RAGE and MCP-1 and increased secretion of MCP-1, sICAM-1 and sVCAM-1 proteins. The half-life for MG reacting with cell protein, assuming a rate constant k_MG, Arg_, is 5.5 × 10^−5^ μM^−1^day^−1^ and arginine residue content in endothelial cells of *approx.* 30 mM^24^, is *approx.* 10 h under physiological conditions and hence the 48 h incubation of HAECs in this study is sufficient for the accumulated MG to react with cell protein and produce the observed inflammatory responses. Increased expression of inflammatory markers, sICAM-1, sVCAM-1 and MCP-1, were found induced by GLO1-knockdown in an immortalised HUVEC-derived cell line, ECRF-24 cells, without proving MG increase and subsequent proteome or collagen expression analysis^6^. Our findings may clearly be linked to and increased MG content and can be linked to coronary artery disease as endothelial inflammation is a driver of endothelial dysfunction and subsequent arteriosclerosis. Taking into account the very recent findings of Xue *et al*. that for overweight and obese patients, trans-resveratrol and hesperetin coformulation increases glyoxalase 1 expression and produces improvements in metabolic and vascular health, the contribution of glyoxalase 1 and methylglyoxal in the vascular system becomes obvious in humans^45^.

We further analysed expression levels of eNOS and phosphorylation status of eNOS at amino acids Ser1177, Ser617 and Thr495. Our results indicate that the effect on endothelial function might not be directly linked to the altered expression or phosphorylation of eNOS. Endothelin-1 (ET-1) is produced primarily by vascular endothelial cells and acts to regulate vascular tone via its two cognate receptors, ET-a, which is expressed in smooth muscle cells, and ET-b, which is present on endothelial cells. In mouse aortic endothelial cells MGO administration in form of a pre-treatment impaired insulin-dependent NO production in endothelial cells. Pre-treatment with MGO for 16 h suppressed the effect of insulin on the phosphorylation of the eNOS sites. Consistent with eNOS activation, the insulin-induced NO release in the culture medium of MAECs was suppressed by 16 h MGO pre-treatment. Concomitant with this, the pre-treatment with MGO for 16 h significantly increased the ET-1 release in the culture medium of MAEC cells^46^. These experiments are in line with our observation regarding the methylglyoxal enrichment via GLO1-knockdown. These results suggest that MGO regardless whether given extracellularly (MAEC-system) or accumulated by GLO1-knockdown (our model) causes an imbalance between NO and ET-1 production, which facilitates vasoconstriction. As discussed by Nigro *et al*., NO may contribute to the activation of endothelin-converting enzyme-1 and the subsequent release of ET-1^46^.

Apoptosis is present in our model as shown by annexin staining, although the analysis of caspase-3/7 activities and expressions of the caspases 3 and 7 indicate no significant increase in apoptotic activity on the levels of caspases 3 and 7. Annexin protein levels are increased 1.2 fold in the proteome analysis which also indicates increased apoptosis following GLO1-knockdown.

Figarola *et al*. were able to show that in human umbilical vascular endothelial cells direct stimulation with 0.4 mM MG induced apoptosis and increased caspase 3 and caspase 9 cleavage after an incubation period of 24 h. This effect was reversible by preincubation with an AGE-inhibitor (LR90)^47^. The MG concentrations used in this assay are 1000fold higher than observed in our assays after 72 h of Glo1-knockdown (Fig. 2, concentration MG in cell culture medium). This may account for the more rapid and more pronounced effect of MG on apoptosis, especially in caspase 3 activation. We were able to detect a significant 1.7fold increase in annexin-positive cells following GLO1-knockdown (if compared to WT25mM+NT-siRNA) which is lower than the 5fold increase detected by Figarolo *et al*.^47^. Also we were not able to follow caspase activation, as described by the authors. Taking into account that under GLO1-knockdown the caspase 3 activity is reduced, and PARP cleavage and annexin-presence is elevated, one might assume an accelerated apoptosis in GLO1-knockdown. This hypothesis needs to be further analysed in future experiments with a more detailed view on apoptosis related pathways.

In conclusion, we have shown that the HAEC proteome is impaired by GLO1-knockdown mediated MG accumulation. Proteomic analysis identified targets that might contribute to the development of cardiovascular complications by modifying structural components of the vascular system. Impaired collagen expression and modification as well as lack of vimentin result in destabilisation of endothelial cells and integrity, provoking endothelial dysfunction which we were able to detect. This dysfunction of HAECs likely contributes to vascular dysfunction and underlies links of hyperglycaemia and GLO1 to risk of CVD.

## Additional Information

**How to cite this article**: Stratmann, B. *et al*. Glyoxalase 1-knockdown in human aortic endothelial cells – effect on the proteome and endothelial function estimates. *Sci. Rep.* **6**, 37737; doi: 10.1038/srep37737 (2016).

**Publisher's note:** Springer Nature remains neutral with regard to jurisdictional claims in published maps and institutional affiliations.

## Supplementary Material

Supplementary Information

## Figures and Tables

**Figure 1:**
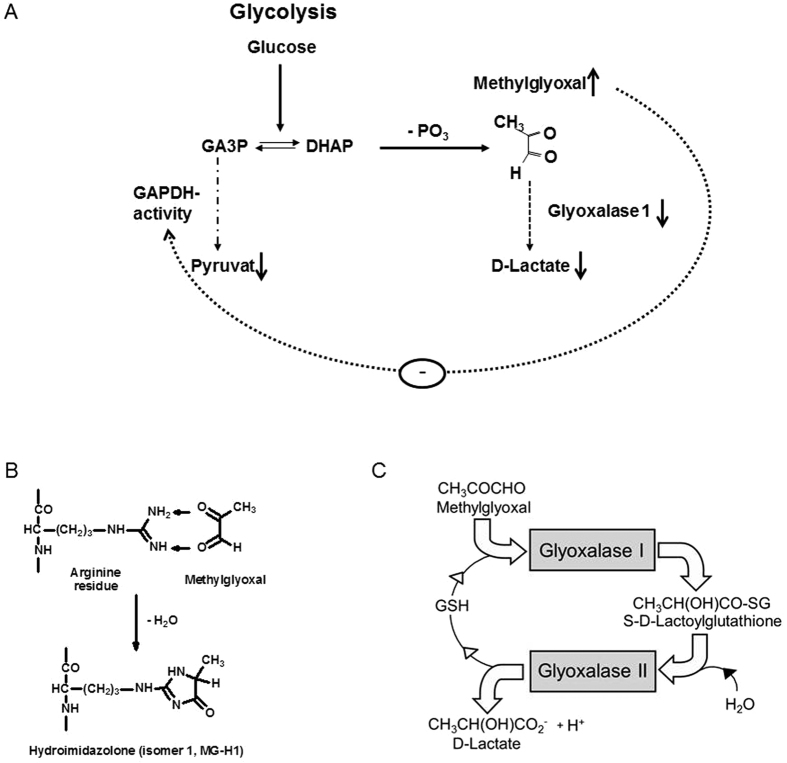
Methylglyoxal, AGE formation and the glyoxalase system. (**A**) Glycolytic pathways leading to MG production and feedback mechanisms of MG on glycolytic pathways. (**B**) Formation of hydroimidazolone MG-H1 by glycation of arginine residues with MG. (**C**) Metabolism of MG by the glyoxalase system.

**Table 1:** Protein glycation by methylglyoxal in human aortal endothelial cells – effect of high glucose concentration and GLO1-knockdown.

Study group	MG-H1 residue (mmol/mol arg)	MG-H1 free adduct (pmol/day/10^6^ cells)
Compartment	Cell protein	Culture medium
5 mM Glucose	0.993 ± 0.088	0.38 ± 0.06
25 mM Glucose	0.956 ± 0.107	0.86 ± 0.22*
25 mM Glucose + NT-siRNA	0.822 ± 0.092	1.02 ± 0.23**
25 mM Glucose + Glo1-siRNA	0.842 ± 0.093	3.17 ± 0.77**^,§§^

**Table 2:** Protein changes after GLO1-knockdown revealed by DIGE/MS analysis (p<0.05).

*Protein*	Gene accession number	fold change	p-value
*LEPREL2* (*Isoform 1 of Prolyl 3-hydroxylase 3*)	IPI00217056.2	1.40 ↓	0.039
*PLOD2 (Procollagen lysine oxoglutarate dehydrogenase 2*)	IPI00945245.1	1.43 ↓	0.038
*LMNA* (*Isoform A of Lamin-A/C*)	IPI00021405.3	1.74 ↓	0.022
*LMNA* (*Isoform ADelta10 of Lamin-A*/*C*)	IPI00216953.1	1.74 ↓	0.022
*DPYSL2* (*Dihydropyrimidinase-related protein 2*)	IPI00257508.4	1.74 ↓	0.022
*VIM* (*Vimentin*)	IPI00418471.6	2.11 ↓	0.027
*TUBA1B* (*TUBA1B protein*)	IPI00793930.1	2.11 ↓	0.027
*Elongation factor 1-alpha*	IPI00940393.1	1.46 ↓	0.034
*HNRNPA1* (*Isoform 2 of Heterogeneous nuclear ribonucleoprotein A1*)	PI00797148.1	1.49 ↓	0.003

Lysates of HAECs transfected with specific GLO1-siRNA or unspecific siRNA control were analysed and protein names assigned as described in Table 2. Presented are fold changes of spot volumes between siRNA control and GLO1-knockdown, ↓ indicates downregulation.
