# Supplementary material for: Glyoxalase 1-knockdown in human aortic endothelial cells – effect on the proteome and endothelial function estimates
Source: Sci Rep. 2016 Nov 29;6:37737. doi: 10.1038/srep37737 (PMC5127188; doi:10.1038/srep37737)
Supplement: Supplementary Information [file srep37737-s1.doc]

**Supplemental 1 to:**

**Glyoxalase 1-knockdown in human aortic endothelial cells – effect on the proteome and endothelial function estimates**

***Running title:*** *Methylglyoxal accumulation in HAECs by GLO1-KD*

Bernd Stratmann^1^, Britta Engelbrecht^1^, Britta C. Espelage^1^, Nadine Klusmeier^1^, Janina Tiemann^1^, Thomas Gawlowski^1^, Yvonne Mattern^1^, Martin Eisenacher^2^, Helmut E. Meyer^2^, Naila Rabbani^3^, Paul J. Thornalley^3^, Diethelm Tschoepe^1^, Gereon Poschmann^2,4*^, Kai Stühler^2,4,5*^

Used antibodies and references citing work

All antibodies used were validated for use in Western Blotting by provider. Most of the antibodies used have been used in published work. References citing used antibodies:

Anti-GLO1 antibody Cat. No. ab96032, provided by abcam, Cambridge , Anti-PLOD2 antibody Cat 21214-1-AP, provided by proteintech, Chicago , Anti-LEPREL2 antibody Cat 16023-1-AP, provided by proteintech, Chicago , Anti-DPYSL2 antibody Cat LS-B3616, provided by LSBio, Seattle, Anti-Caspase-3 antibody Cat 9662, provided by Cell Signaling, MA , Anti-PARP antibody Cat No. 9542, provided by Cell Signaling , Anti-Col1A1 antibody Cat No. LS-C150353, provided by LSBio , Anti-Col5A1 antibody Cat AF6110, provided by AcrisAntibodies, Herford , Anti-Total eNOS antibody Cat ab76198, provided by abcam , Anti-eNOS pT494 antibody Cat No. ab138430, provided by abcam , Anti-eNOS pS615 antibody Cat. No. ab138458, by abcam , Anti-eNOS pS1177 antibody Cat. No. ab184154, by abcam , Anti-ß-actin antibody Cat No. 3700, provided by Cell Signaling , Anti α-tubulin antibody Cat No. 2125 provided by Cell Signaling

1. Shan, G., et al., *Increase in Blood Glutathione and Erythrocyte Proteins Related to Glutathione Generation, Reduction and Utilization in African-American Old Women with Diabetes.* J Sci Technol Environ, 2015. **5**(1).

2. Cabral, W.A., et al., *Absence of the ER Cation Channel TMEM38B/TRIC-B Disrupts Intracellular Calcium Homeostasis and Dysregulates Collagen Synthesis in Recessive Osteogenesis Imperfecta.* PLoS Genet, 2016. **12**(7): p. e1006156.

3. Heard, M.E., et al., *Sc65-Null Mice Provide Evidence for a Novel Endoplasmic Reticulum Complex Regulating Collagen Lysyl Hydroxylation.* PLoS Genet, 2016. **12**(4): p. e1006002.

4. Shen, J., et al., *Vascular-targeted TNFalpha and IFNgamma inhibits orthotopic colorectal tumor growth.* J Transl Med, 2016. **14**(1): p. 187.

5. Reese, C., et al., *Fibrocytes in the fibrotic lung: altered phenotype detected by flow cytometry.* Front Pharmacol, 2014. **5**: p. 141.

6. Papaconstantinou, I., et al., *Effect of infliximab on the healing of intestinal anastomosis. An experimental study in rats.* Int J Surg, 2014. **12**(9): p. 969-75.

7. Cobley, J.N., et al., *Lifelong training preserves some redox-regulated adaptive responses after an acute exercise stimulus in aged human skeletal muscle.* Free Radic Biol Med, 2014. **70**: p. 23-32.

8. Li, P., et al., *Uric acid enhances PKC-dependent eNOS phosphorylation and mediates cellular ER stress: A mechanism for uric acid-induced endothelial dysfunction.* Int J Mol Med, 2016. **37**(4): p. 989-97.

9. Tran, Q.K., et al., *Estrogen Enhances Linkage in the Vascular Endothelial Calmodulin Network via a Feedforward Mechanism at the G Protein-coupled Estrogen Receptor 1.* J Biol Chem, 2016. **291**(20): p. 10805-23.

10. Du, L., et al., *eNOS/iNOS and endoplasmic reticulum stress-induced apoptosis in the placentas of patients with preeclampsia.* J Hum Hypertens, 2016.

11. Li, Q., et al., *Stromal cell-derived factor-1 promotes human adipose tissue-derived stem cell survival and chronic wound healing.* Exp Ther Med, 2016. **12**(1): p. 45-50.

12. Churchward-Venne, T.A., et al., *Citrulline does not enhance blood flow, microvascular circulation, or myofibrillar protein synthesis in elderly men at rest or following exercise.* Am J Physiol Endocrinol Metab, 2014. **307**(1): p. E71-83.
